# The Systemic Response to Topical Aldara Treatment is Mediated Through Direct TLR7 Stimulation as Imiquimod Enters the Circulation

**DOI:** 10.1038/s41598-017-16707-5

**Published:** 2017-11-29

**Authors:** Louis Nerurkar, Alison McColl, Gerard Graham, Jonathan Cavanagh

**Affiliations:** 1 University of Glasgow, Institute of Infection, Immunity & Inflammation, G12 8TA Glasgow, Scotland; 2 University of Glasgow, Institute of Health & Wellbeing, G12 8TA Glasgow, Scotland

## Abstract

Topical application of Aldara cream, containing the Toll-like receptor 7/8 agonist Imiquimod, is a widely used mouse model for investigating the pathogenesis of psoriasis. We have previously used this model to study the effects of peripheral inflammation on the brain, and reported a brain-specific response characterised by increased transcription, infiltration of immune cells and anhedonic-like behavior. Here, we perform a more robust characterisation of the systemic response to Aldara application and find a potent but transient response in the periphery, followed by a prolonged response in the brain. Mass spectrometry analysis of plasma and brain samples identified significant levels of Imiquimod in both compartments at molar concentrations likely to evoke a biological response. Indeed, the association of Imiquimod with the brain correlated with increased Iba1 and GFAP staining, indicative of microglia and astrocyte reactivity. These results highlight the potency of this model and raise the question of how useful it is for interpreting the systemic response in psoriasis-like skin inflammation. In addition, the potential impact on the brain should be considered with regards to human use and may explain why fatigue, headaches and nervousness have been reported as side effects following prolonged Aldara use.

## Introduction

Aldara cream is most commonly used for the topical treatment of external genital warts, actinic keratosis and superficial basal cell carcinoma. Its active component, Imiquimod (IMQ), is a TLR7/8 agonist that induces a potent anti-viral response characterised by the production of type I interferons (IFN), pro-inflammatory cytokines and chemokines^1^. Its clinical use highlighted common local side effects including erythema, swelling, scabbing and itching of the skin^2,3^ and it was subsequently tested in rodents as a potential model of psoriasis. As such, the Aldara model of psoriasis-like skin inflammation was first described in 2009 by Van Der Fits *et al*. and has since become one of the most widely used models in preclinical psoriasis studies^4^.

Since 2009, many studies have examined the pathogenesis of this model at the site of application. It is now thought that the response to Aldara cream is mediated primarily through CD4^+^ T cells, specifically Th17 cells and intradermal γδ-T cells in an IL-17/IL-23-dependent manner, much like human psoriasis^4,5^. The skin response is characterised by local type I IFN and pro-inflammatory cytokine production, immune cell infiltration into the skin and hyperkeratosis.

In addition to the local skin response, topical Aldara application was noted to cause systemic pro-inflammatory cytokine production and enlarged spleen and draining lymph nodes^4,6^. As such, as well as its use for studying psoriasis-like inflammation in the local tissue, the Aldara model has been used to investigate the systemic effects of psoriatic inflammation. For example, Hackstein *et al*. used topical Aldara treatment to examine the effects of skin TLR7 antagonism on respiratory leukocyte populations^7^. Alongside them, Al-Harbi *et al*. have used this model to demonstrate psoriasis-induced hepatic inflammation^8^. In addition, we have previously used the Aldara model to investigate the systemic effects of IMQ and TLR7 activation, with a particular focus on the distal brain response^6,9^. We have shown that topical Aldara treatment induced a transcriptional interferon stimulated gene (ISG) and chemokine response in what appeared to be a brain-specific manner, along with the infiltration of immune cells into the brain, a reduction in hippocampal neurogenesis and a reduction in burrowing behaviour.

The precise mechanisms underpinning this brain response were of great interest to us, as many potential routes of communication exist between the periphery and the brain^10^. For example, IMQ could activate TLR7 on peripheral nerves, transmitting the inflammatory signal through the neural pathway^11^. Alternatively, pro-inflammatory cytokines and other soluble mediators could enter the circulation and reach the brain either by active transport across, or disruption of, the blood-brain-barrier^10^. Finally, if IMQ was able to enter the circulation and the brain directly, several brain-resident cells types including microglia, astrocytes and neurons, have been shown to express TLR7 and would therefore be able to respond directly^12,13^.

A growing literature clearly points towards the existence of a systemic response following topical Aldara treatment, however this was not particularly apparent in our previous studies. Whilst the PBL response was investigated alongside the brain as an indicator of systemic inflammation, the extent of the response was difficult to assess as we did not characterise a range of peripheral tissues or early time-points. Therefore, the aim of this study was to systematically characterise the tissue response to Aldara treatment across a detailed time-course model to determine the most likely mechanism driving systemic inflammation. An early transcriptional response throughout all peripheral tissues prompted further investigation regarding the capacity of IMQ to enter the circulation, and subsequently the brain. Our findings, presented in this current study, show IMQ is present in both the plasma and brain as early as 4 hours after treatment, suggesting the primary mechanism of immune activation of topical Aldara treatment is through the direct ligation of IMQ with TLR7 receptors throughout the body.

## Results

### Topical Aldara application induces a transcriptional chemokine and cytokine response in the brain as early as four hours after treatment

**Figure 1:**
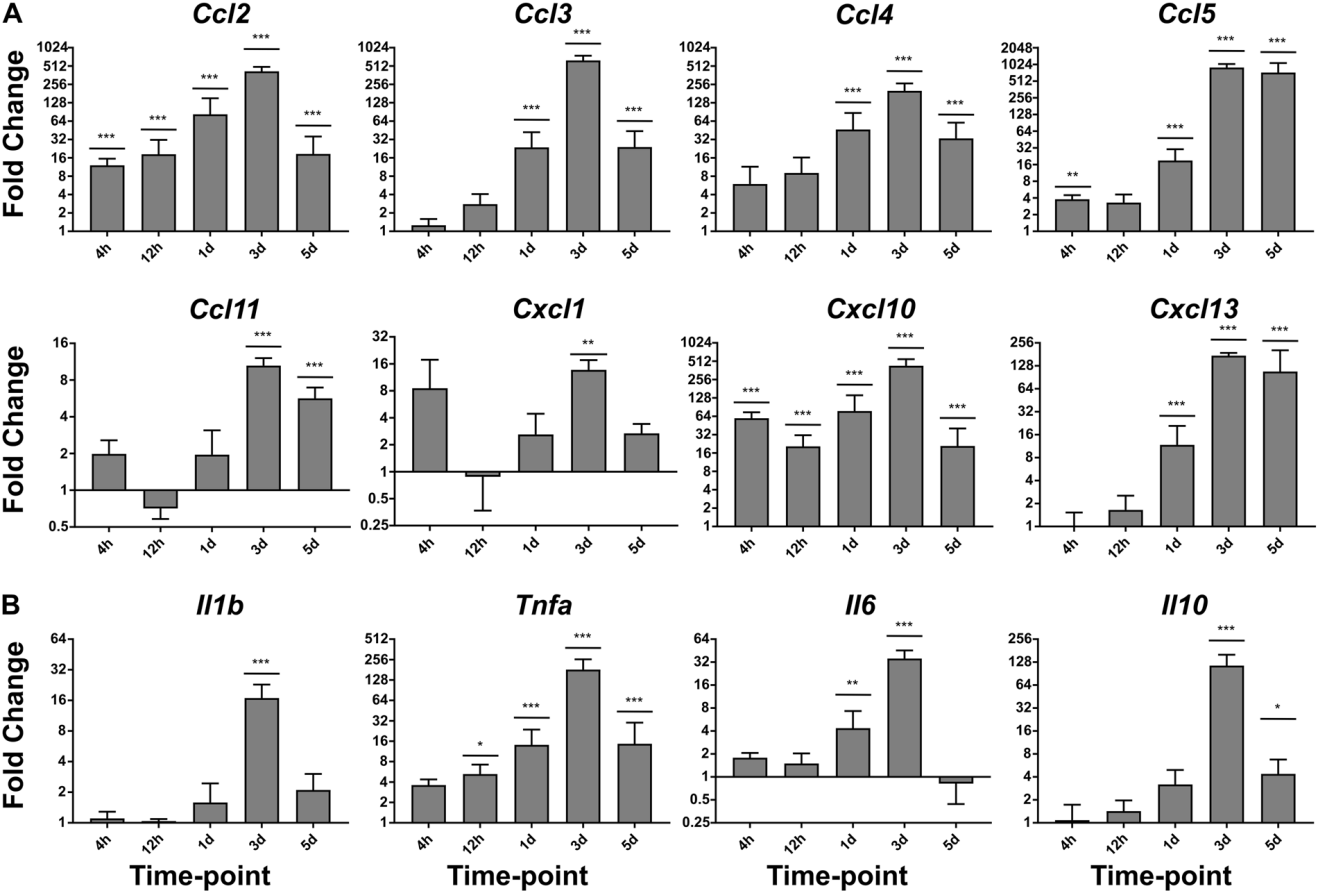
Topical Aldara application induces a chemokine and cytokine transcriptional response in the brain as early as 4 hours after treatment. QRT-PCR analysis was performed on RNA from perfused brain samples from mice treated daily with Aldara or control cream. Samples were collected 4 hr, 12 hr, 1 d, 3 d and 5 d after the initial treatment. The expression profile of 8 chemokines (**A**) and 4 cytokines (**B**) was assessed. Expression values were normalized to housekeeping gene *Tbp*. Data are presented as mean (+/−SD) fold changes vs respective controls. N = 4 mice per group. Data were log transformed and significance was determined using one-way ANOVA with post-hoc Student’s t-test with Bonferroni’s multiple testing correction. *P < 0.05; **P < 0.01; ***P < 0.001.

Having previously shown that topical Aldara treatment induces a transcriptional response in the brain that peaks 3–5 days after treatment, we sought to determine the temporal mechanisms of the brain response. To do so, we performed a detailed time course analysis incorporating 4 hr, 12 hr, 1 day, 3 days and 5 days post-treatment, analysing a panel of 8 chemokines (Fig. 1A) and 4 cytokines (Fig. 1B) using QRT-PCR. The time course model showed that multiple chemokine transcripts were upregulated as early as 4 hr after treatment, including statistically significant increases in levels of *Ccl2*, *Ccl5* and *Cxcl10*. By 1 day, *Ccl3*, *Ccl4* and *Cxcl13* were also significantly raised in the brain when compared with controls. In keeping with our previous findings, 3 days appeared to be the peak of the transcriptional response where all chemokines were significantly upregulated. Between 3 and 5 days, this response began to ameliorate, however the expression of all but *Cxcl1* remained significantly higher in the brains of Aldara treated mice at day 5. With regard to the cytokine transcripts (Fig. 1B), *Tnfa* appeared to be the earliest induced in the brain, with a significant increase from 12 hr after treatment through to 5 days. *Il6* was significantly induced 1 day after treatment and by 3 days *Il1b*, *Tnfa*, *Il6* and *Il10* were all significantly upregulated in the brains of treated mice. Interestingly *Il10*, a cytokine considered to mediate anti-inflammatory effects, did not significantly increase until 3 days, but remained significantly elevated at 5 days, perhaps indicating a delayed resolution of the brain response. As *Il-17* and *Il-23* have previously been associated with the dermal response to IMQ we also assessed their expression in the brain^4^. *Il-17* was not detectable and *Il-23a* was not found to differ significantly between treated and control groups at any time-point (Supplementary Figure 1).

### The transcriptional chemokine and cytokine response in the brain following topical Aldara treatment is translated into protein

**Figure 2:**
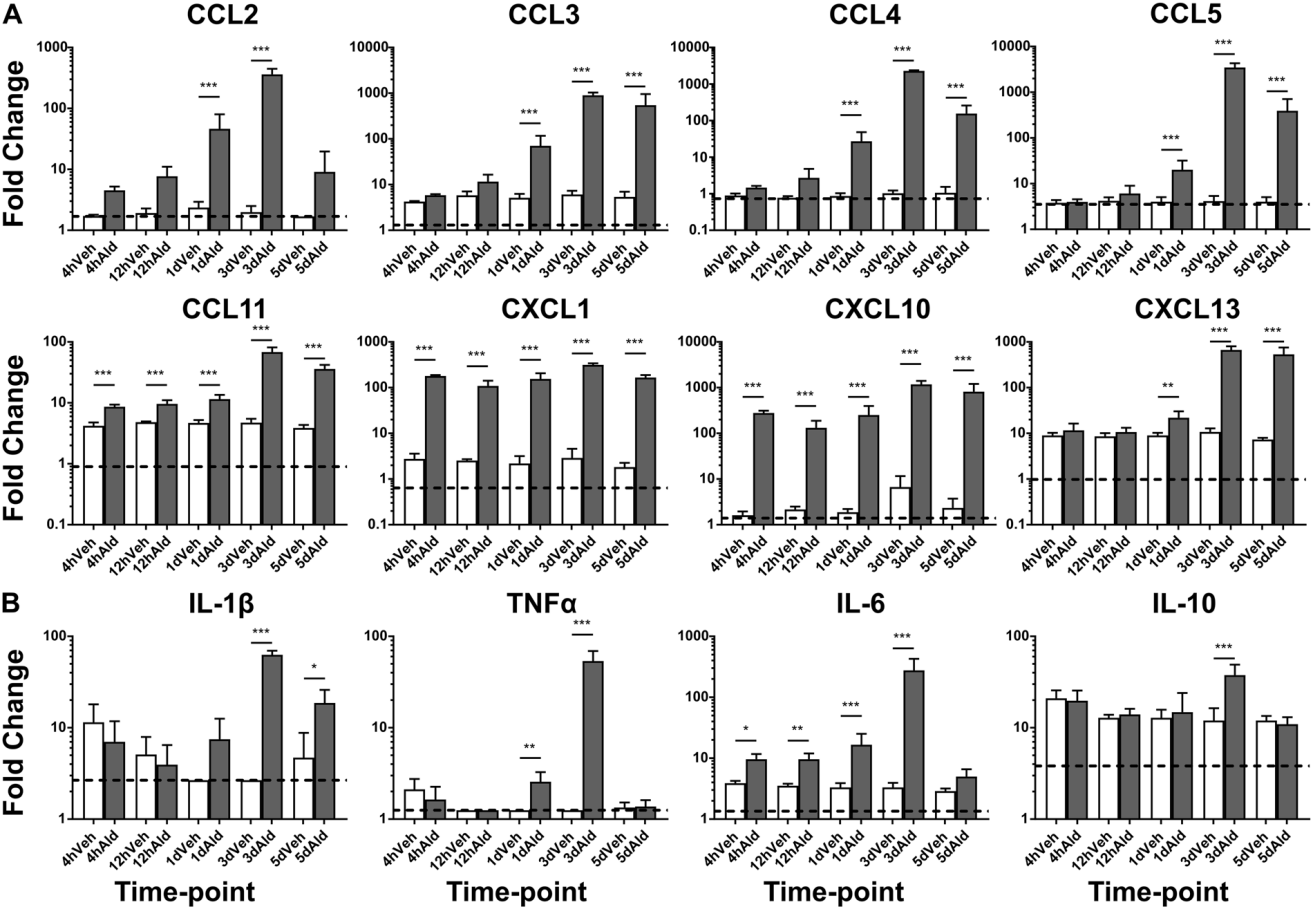
Topical Aldara application induces a chemokine and cytokine protein response in the brain as early as four hours after treatment. Biolegend LegendPlex protein array analysis was performed on perfused brain samples from mice treated daily with Aldara or control cream. Samples were collected 4 hr, 12 hr, 1 d, 3 d and 5 d after initial treatment. The expression profile of 8 chemokines (**A**) and 4 cytokines (**B**) was assessed. Samples below the limit of detection were set to the limit of detection. N = 4 mice per group. Data are presented as mean (+/−SD) of absolute protein values. Data were log transformed and significance was determined using one-way ANOVA with post-hoc Student’s t-test with Bonferroni’s multiple testing correction. *P < 0.05; **P < 0.01; ***P < 0.001.

Topical Aldara treatment induced a marked increase in a number of chemokine and cytokine gene transcripts in the brain throughout the time course model. To determine the biological relevance of this response, protein levels of 8 chemokines and 4 cytokines were measured in brain homogenate (Fig. 2A and B, respectively) 4 hr, 12 hr, 1 day, 3 days and 5 days after treatment. CCL11, CXCL1 and CXCL10 displayed significantly elevated levels of expression in the brain at 4 hr and 12 hr after Aldara treatment when compared with controls. By 1 day, all chemokines analysed, including CCL2, CCL3, CCL4, CCL5 and CXCL13, were significantly upregulated in the brains of treated mice. These remained raised until day 3, at which point several of the chemokines were being expressed at levels between 100–1000 pg/ml. By 5 days, the protein levels tended to decrease, however CCL3, CCL4, CCL5, CCL11, CXCL1, CXCL10 and CXCL13 remained significantly higher than control levels. With regards to cytokine expression, IL-6 was the first to be upregulated in the brain and was significantly induced at 4 hr and 12 hr. By 1 day, TNFα was also significantly upregulated in the brains of Aldara treated mice. The highest level of induction of cytokines occurred at day 3, when all cytokines analysed were significantly induced, however by day 5, IL-1β was the only cytokine to remain significantly raised in the treated mice. In addition to the cytokines shown in Fig. 2B, the protein assay allowed us to measure IFNβ and IFNγ in the brain, both of which were significantly induced at day 3 (Supplementary Figure 2). Surprisingly, the protein expression of CCL11 and CXCL1 was significantly induced at all time-points despite this increase not being reflected at the transcript level.

### Topical Aldara application induces a transcriptional response in systemic tissues that is temporally unique compared with the brain

**Figure 3:**
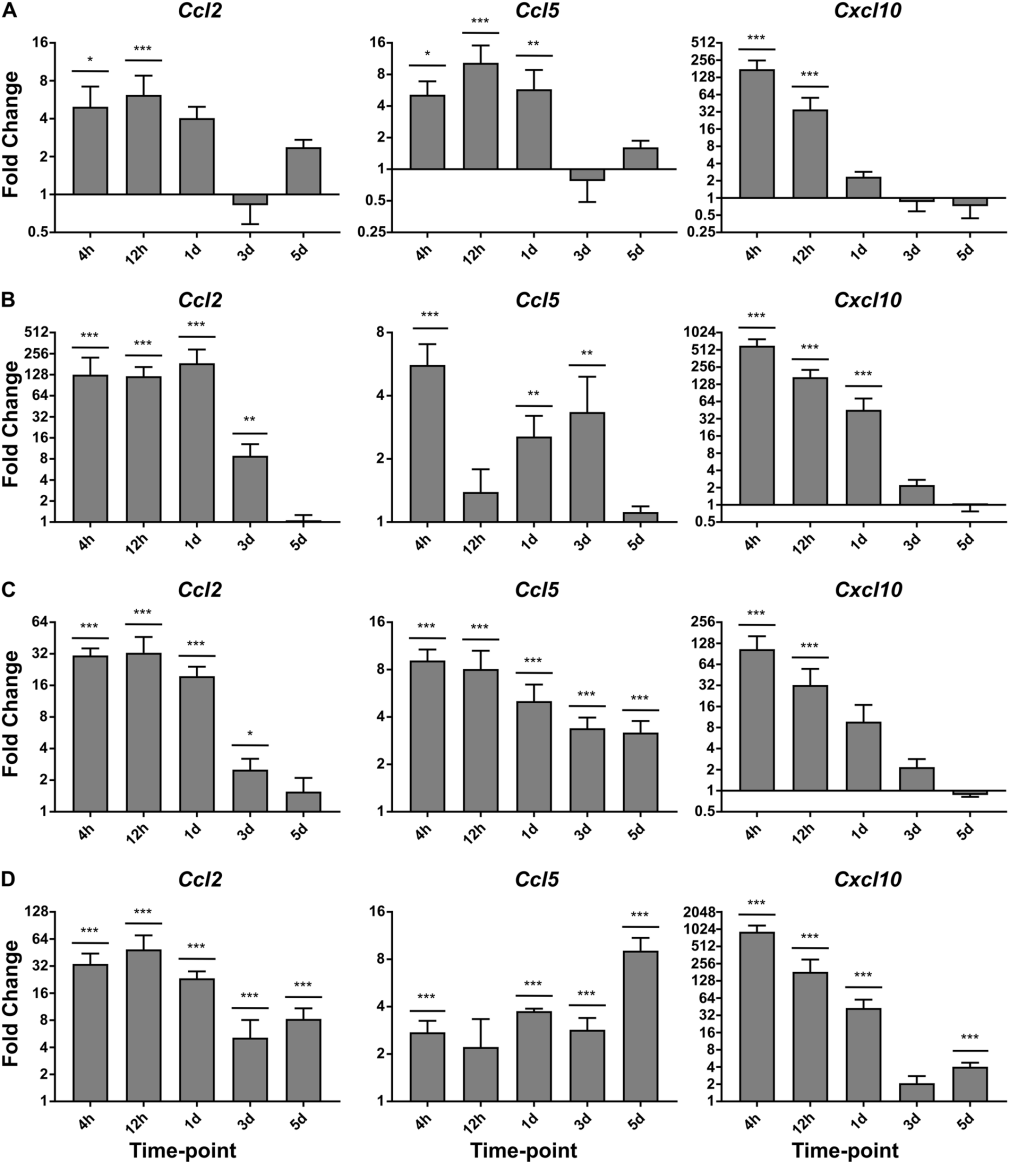
Topical Aldara application induces a chemokine transcriptional response at the local site of application and within the PBLs, Lungs and Liver. QRT-PCR analysis was performed on PBLs and perfused skin, lung and liver samples from mice treated daily with Aldara or control cream. Samples were collected 4 hr, 12 hr, 1 d, 3 d and 5 d after the initial treatment. The expression profile of 3 chemokines was assessed in (**A**) Skin (**B**) PBL (C) Liver and (D) Lungs. Expression values were normalised to housekeeping gene *Tbp*. Data are presented as mean (+/−SD) fold changes vs respective controls. N = 4 mice per group. Data were log transformed and significance was determined using one-way ANOVA with post-hoc Student’s t-test with Bonferroni’s multiple testing correction. *P < 0.05; **P < 0.01; ***P < 0.001.

**Figure 4:**
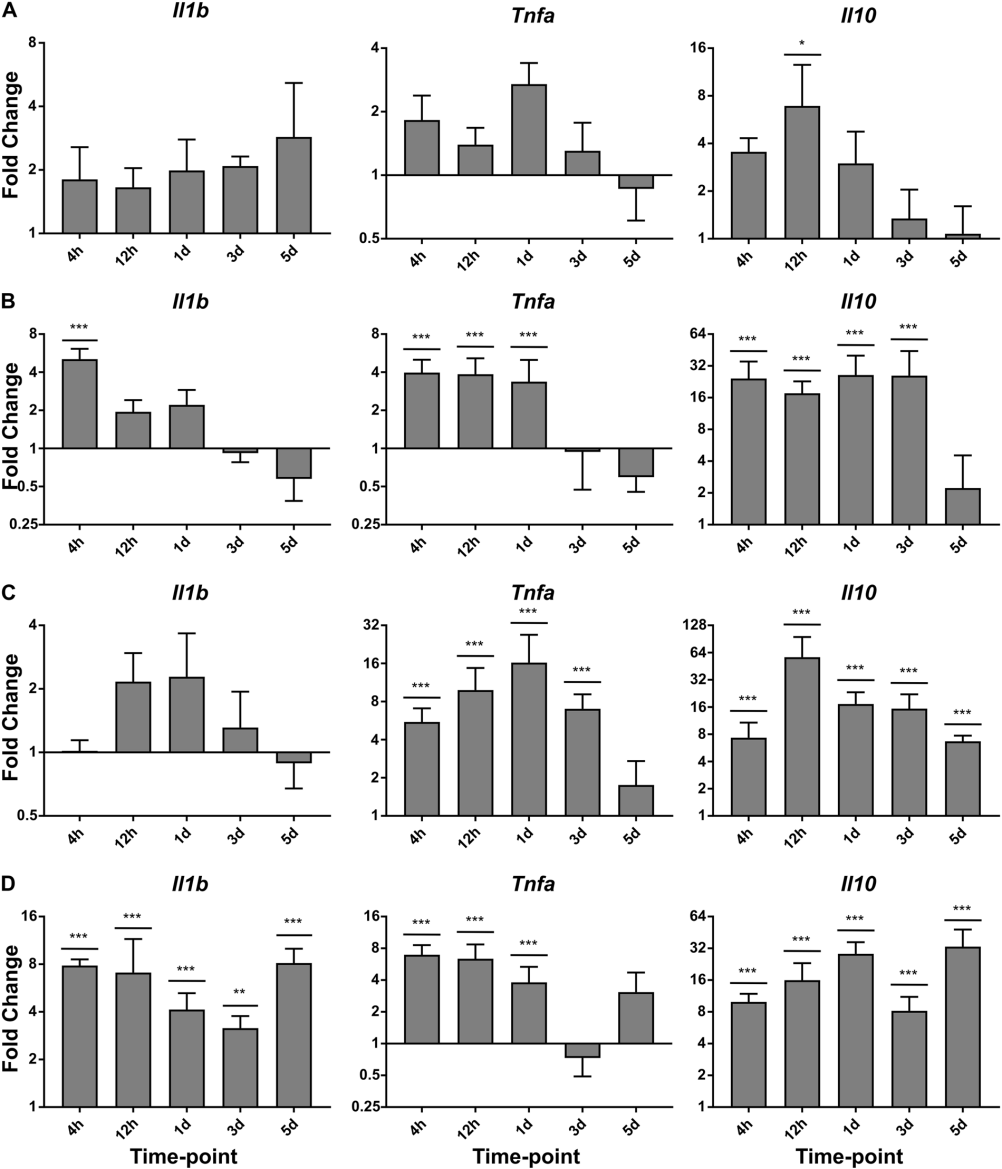
Topical Aldara application induces a cytokine transcriptional response at the local site of application and within the PBLs, Lungs and Liver. QRT-PCR analysis was performed on PBLs and perfused skin, lung and liver samples from mice treated daily with Aldara or control cream. Samples were collected 4 hr, 12 hr, 1 d, 3 d and 5 d after the initial treatment. The expression profile of 3 cytokines was assessed in (**A**) Skin (**B**) PBL (**C**) Liver and (**D**) Lungs. Expression values were normalised to housekeeping gene *Tbp*. Data are presented as mean (+/−SD) fold change vs respective controls. N = 4 mice per group. Data were log transformed and significance was determined using one-way ANOVA with post-hoc Student’s t-test with Bonferroni’s multiple testing correction. *P < 0.05; **P < 0.01; ***P < 0.001.

In previous studies we have compared the brain response with the peripheral blood leukocyte (PBL) response as an indicator of systemic inflammation following Aldara treatment. The results showed very little upregulation of expression, however the earliest time point investigated was 24 hr. To determine if Aldara treatment induces an early systemic response, we assessed a number of peripheral organs, including the skin (A), PBL (B), liver (C) and lungs (D), at 4 hr, 12 hr, 24 hr, 72 hr and 120 hr post-treatment. We have focused on three classic inflammatory chemokines, Ccl2, Ccl5 and Cxcl10 (Fig. 3A–D), and three archetypal cytokines Il1b, Tnfa and Il10 (Fig. 4A–D), although a more detailed panel of genes were investigated (Supplementary Figures 3–6). Both the PBL and the lungs exhibited significant upregulation of *Ccl2*, *Ccl5*, *Cxcl10*, *Il1b*, *Tnfa* and *Il10* at 4 hours after treatment. *Cxcl10* was the gene with the biggest fold change induction compared to control mice, with a striking 500-fold increase in the PBL and almost 1000-fold increase in the lungs. With the exception of *Il1b*, all genes were significantly upregulated in the liver at 4 hours. The skin displayed the highest degree of variation, although all three chemokines were significantly induced at 4 hours and 12 hours after treatment. Interestingly, *Il1b* and *Tnfa* failed to reach significance despite an upward trend and *Il10* was only significantly higher at 12 hours after treatment. Whilst the peripheral tissues displayed a strong response at the earlier time points, the response tended to ameliorate over the course of the model and by day 3, many cytokines and chemokines were close to control levels.

### Imiquimod enters the circulation and the brain within 4 hours of topical Aldara application

**Figure 5:**
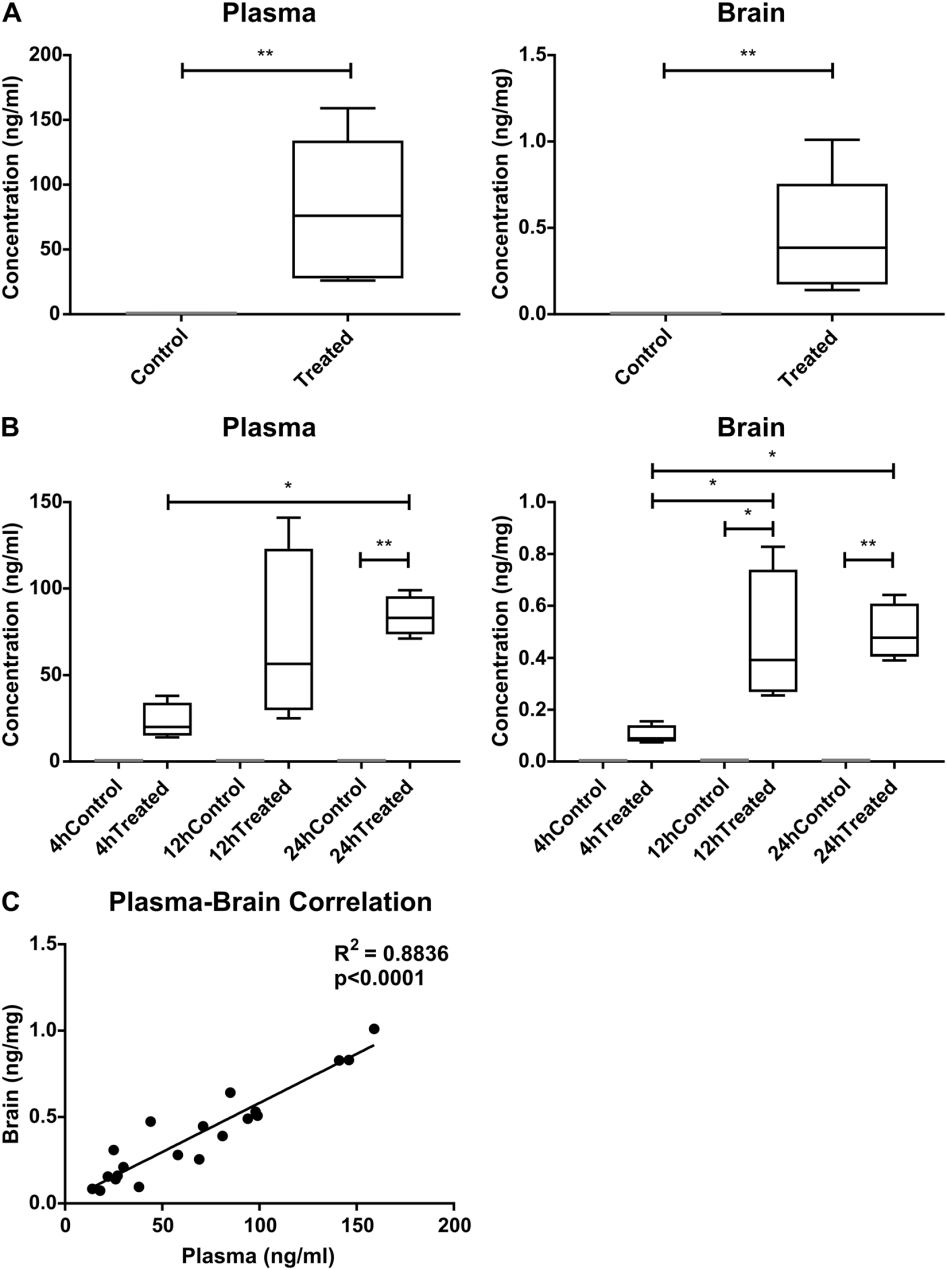
Topical Aldara application results in entry of the TLR7 agonist imiquimod into the plasma and brain of mice as early as four hours after treatment. Mass spectrometry analysis was performed on plasma and perfused brains of mice treated daily with Aldara or control cream. Imiquimod levels were assessed (**A**) 24 hours after 3 daily treatments (N = 8 mice per group) and (**B**) 4 hr, 12 hr and 24 hr after a single treatment (N = 4 mice per group). (**C**) Linear regression analysis on matched plasma and brain samples was performed. Box represents 25–75^th^ percentile and whiskers represent min and max values. Significance was determined using (**A**) Student’s t-test (**B**) One-way ANOVA with post-hoc Student’s t-test with Bonferonni’s multiple testing correction. (**C**) Linear regression analysis. *P < 0.05; **P < 0.01; ***P < 0.001.

Topical Aldara treatment induces a potent transcriptional response throughout peripheral tissues including the skin, PBL, lungs and liver. This was evident within 4 hours of treatment, indicating a very rapid dissemination of the immune response. With this in mind, it was important to determine if the active component, IMQ, was entering the circulation, and subsequently the CNS. To address this question, plasma and perfused brains were extracted from mice treated with Aldara cream for 3 days, in line with the peak transcriptional and protein response in the brain. Results showed that IMQ was present in both the plasma and brain at 3 days, in quantities equal to 0.33 μM (95% CI; 0.15–0.52) in the plasma with a total load of 0.76 nmol (95% CI; 0.31–1.21 nmol) in the brain (Fig. 5A). To determine the temporal nature of IMQ dissemination throughout the plasma and brain, we assessed three earlier time points, 4 hr, 12 hr and 24 hr after treatment. Consistent with the 3 day results, IMQ was present in both compartments at all time points analysed, and reached significant levels at 12 hr, 24 hr and 3 days when compared with levels in control mice (Fig. 5B). Correlation analysis was performed to determine the relationship between plasma levels and brain levels of IMQ, showing a very strong positive relationship with an r^2^ equal to 0.9 (Fig. 5C).

### IMQ entry into the CNS is associated with reactive gliosis

**Figure 6:**
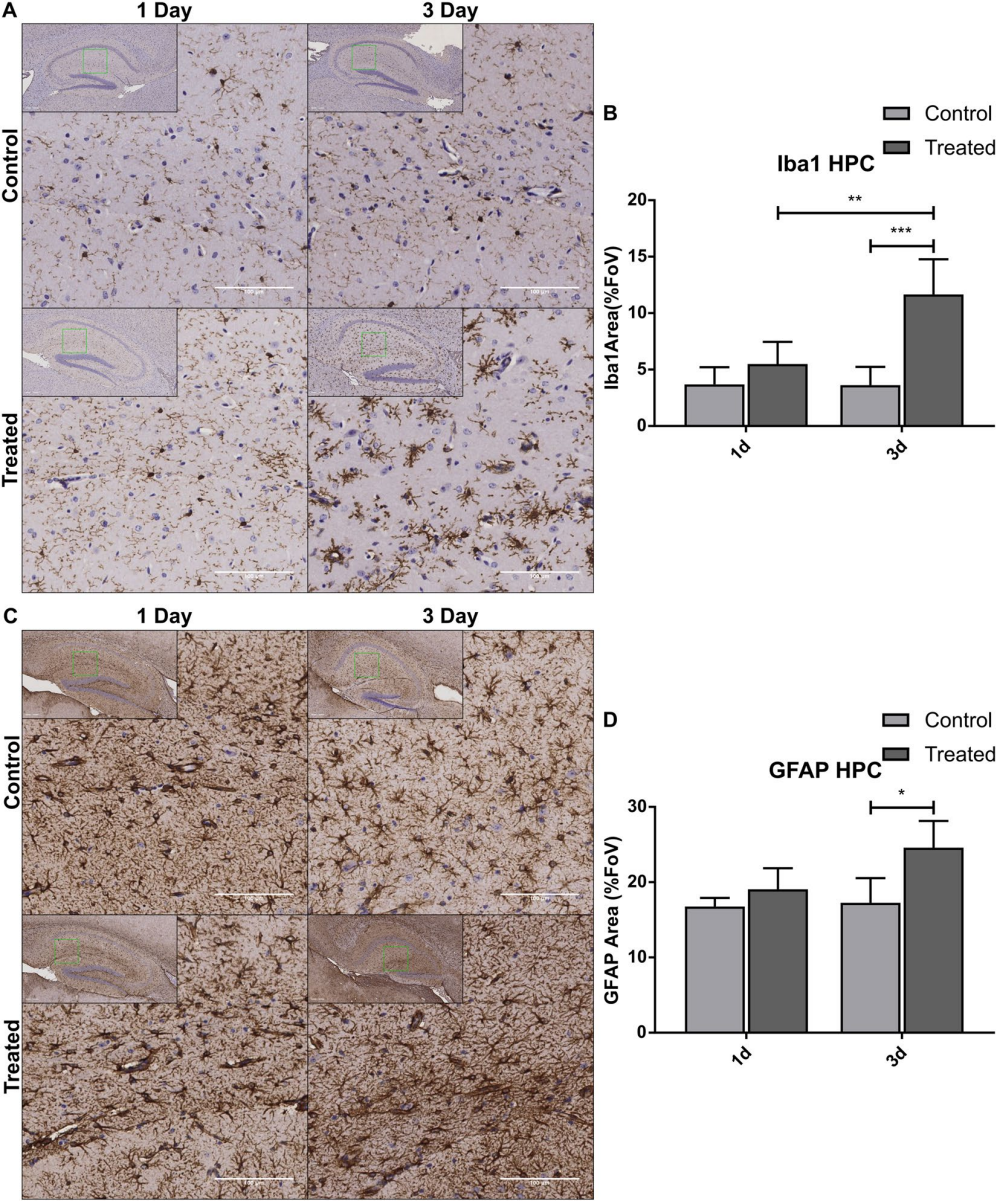
Topical Aldara application induces significant changes in Iba1^+^ and GFAP^+^ cells in the hippocampus. Immunohistochemistry was performed on formalin-fixed paraffin embedded brain sections from mice treated daily with Aldara or control cream. Samples were collected 24 hr after first treatment and 24 hr after 3 daily treatment. (**A**) Representative images of Iba1^+^ staining in mouse hippocampus. (**B**) Quantification of Iba1^+^ area in mouse hippocampus. (**C**) Representative images of GFAP^+^ staining in mouse hippocampus. (**D**) Quantification of GFAP^+^ area in mouse hippocampus. Quantification was performed using ImageJ analysis software. Significance was determined using one-way ANOVA with post-hoc Student’s t-test with Bonferroni’s multiple testing correction. *P < 0.05; **P < 0.01; ***P < 0.001.

We have previously reported that Aldara application is associated with immune cell infiltration into the CNS; however the response of brain-resident immune cells has not yet been evaluated. With the knowledge that IMQ can reach the brain, it was important to determine the response of resident cells that express TLR7 receptors, namely microglia and astrocytes. Using Iba1 and GFAP respectively, the response of microglia and astrocytes was assessed using immunohistochemistry at 1 day and 3 days after application. With regards to the Iba1 staining (Fig. 6A), there were clear morphological differences between the control and treated brains, with treated cells displaying thickened processes and alterations in ramification. As we had previously identified changes in hippocampal neurogenesis in response to Aldara treatment, we chose to quantify Iba1 and GFAP staining in this region. By 3 days after treatment, the Iba1-stained area was significantly greater than in the control brains, indicating significant alterations in the Iba1^+^ population (Fig. 6B). The same was true of GFAP staining at 3 days, which again was significantly higher in the treated brains than in the control brains (Fig. 6C and D).

## Discussion

In this study, we have shown that topical Aldara treatment induces a potent chemokine and cytokine response throughout the peripheral tissues and in the brain, and that these responses are temporally distinct. The active component of Aldara cream, TLR7/8 antagonist IMQ, reaches the plasma and the brain within 4 hours of treatment, and appears to reach equilibrium at around 12 hours post-application. The concentration of IMQ in the brain was positively and significantly correlated with that of the plasma and reached molar concentrations of 0.33 μM in the plasma with a total load of 0.76 nmol in the brain at 3 days. Furthermore, direct entry of IMQ into the brain induces a reactive state of microglia and astrocytes, likely mediated through direct TLR7 activation. Our data suggest that the systemic effects of topical Aldara treatment are mediated through direct TLR7 stimulation.

While the systemic organs showed a rapid response, with significant upregulation of chemokine and cytokine transcripts by four hours, the transcriptional response began to ameliorate quickly and many of the cytokines and chemokines returned to control levels between day 1 and day 3. In contrast, whilst there was a moderate upregulation of some of the genes at the earlier time points, the peak of the brain response was at day 3. This was also true of the protein expression, where day 3 appeared to be the peak of the chemokine and cytokine response in the brain. It is important to mention there was some disparity between measured transcript and protein levels of CXCL1 and CCL11, however it is possible the higher protein induction was the result of the release of pre-stored cytoplasmic granules^14^ or peripheral sources entering the brain, as has been shown for many cytokines^15^. We had previously hypothesised that the delayed brain response may be due to the length of time it takes for the brain to be activated from a distal application site^9^. However, the results of the mass spectrometry would suggest otherwise since detectable levels of IMQ were identified at 4 hours after application. In this study we clearly identified that the response in the brain was more prolonged than that of the periphery. Interestingly, the anti-inflammatory molecule IL-10 is only upregulated in the brain 3 days after the application of Aldara, whereas in 3 out of 4 peripheral tissues examined, IL-10 was significantly induced by 4 hours. Thus one potential explanation for the temporally unique brain response is the delayed onset of pro-resolution factors. Indeed, IL-10 induction is thought to be important for regulatory immunosuppression following TLR7 stimulation^16^ and IL-10-deficient mice showed higher pro-inflammatory cytokine levels following I.P LPS administration^17^.

The results of the mass spectrometry experiment have provided a great deal of clarity regarding the brain response we have previously reported. With IMQ entering the brain so rapidly after treatment, it is likely that brain-resident, TLR7-expressing cells are the primary mediators of the brain response. This is particularly true considering earlier work has demonstrated that significant recruitment of immune cells to the CNS does not appear to occur within the first 24 hours^9^. This direct activation would explain the type I IFN response we have observed at both the transcriptional and protein levels and allows us to understand how other reported brain changes may have manifested. Aldara application induced protein expression of IFNb and IFNg in the brain between 1 and 3 days after treatment. In addition, protein levels of pro-inflammatory cytokines IL-1b and TNFa were significantly increased in the brain, both of which, along with IFNg, are known to activate IDO, facilitating the breakdown of the serotonin precursor, tryptophan. These findings are interesting in the context of previously identified reductions in burrowing behaviour, an indicator of anhedonia, as cytokines are known to induce sickness behaviours in rodents^9,18^. In addition, cytokines can decrease brain-derived neurotrophic factor (BDNF), adversely affecting neurogenesis and neuroplasticity^19,20^. Again, this is interesting in the context of our previous findings, as we have shown that topical Aldara treatment is associated with a reduction in hippocampal neurogenesis.

While research into brain specific effects of TLR7 is limited, Butchi *et al*. investigated the brain response to TLR stimulation using intracerebroventricular (i.c.v) injection of IMQ directly into the CNS of neonates^21,22^. They performed a dose response using concentrations of IMQ ranging from 20nmol per mouse to 500nmol per mouse and observed an upregulation of inflammatory cytokines and chemokines at all doses, 12 hours after injection. Whilst the response to TLR7 agonists was not nearly as potent as other TLR ligands, including CpG and LPS, IMQ injection induced a cellular response characterised by infiltrating leukocytes and the activation of brain-resident glial cells. Interestingly, Butchi *et al*. administered considerably higher doses of IMQ into the brain than we would have achieved using the topical Aldara model; however the response we see is arguably more extensive than that which they have reported. This may be due to an exaggerated immune response in the brain when accompanied by peripheral immune stimulation, a phenomenon sometimes referred to as ‘priming’^23^. Evidence of this has been shown in rodent models of prion disease and aged mice, where additional LPS stimulation exacerbates CNS inflammation by sensitising immune cells^24,25^. In addition, in early skin graft studies of immune privilege within the CNS, peripheral immune stimulation was shown to be a key event for inducing effective CNS immune responses^26^.

Aldara cream is used clinically for the treatment of genital warts, small superficial skin cancers and actinic keratosis, mimicking a viral infection to induce a potent anti-viral response. Whilst the dose for human use is significantly lower by body weight than that used in this mouse model, it is important to consider the potential consequences of long term use with the knowledge that IMQ has the ability to reach the CNS in rodents. Whilst it is not possible to assess the capacity of IMQ to enter the human brain, studies have shown that absorption through the skin can lead to measurable concentrations of IMQ in both the serum and urine^27^. Indeed, there are a number of case studies demonstrating that IMQ treatment can induce unintended systemic side effects that range in severity and frequency, including fever, fatigue, headaches and raised erythrocyte sedimentation rate^28^. Bearing in mind these systemic effects, and taking into consideration the small molecular weight (240.3 g/mol) and lipophilic nature of IMQ, it is feasible that circulating IMQ could enter the human brain with similar efficiency as it does in rodents. One consideration for this rodent model is the possibility of an oral route of absorption as described by Grine *et al*. However, considering that IMQ has been detected in human pharmacological studies where IMQ will not have been ingested we propose that, although this may contribute, topical absorption will occur and allow entry to the bloodstream regardless of oral ingestion of Aldara cream.

A considerable challenge in neuroimmunology is the ability to introduce immune stimuli into the CNS using non-infectious agents in a non-invasive manner. In this study we have identified that topically applied IMQ can directly enter the CNS and, as such, this provides an alternative tool for the study of neuroimmune responses to TLR stimulation that does not require i.c.v injections. In addition, as it is introduced through a peripheral route, it is likely to be more biologically relevant than i.c.v injections that are generally considered to be a non-physiological method of introducing agents into the CNS. The results presented in this study highlight the need for greater caution when interpreting the response to topical IMQ treatment, particularly in the context of psoriasis.

## Methods

### Animals

Female C57BL/6 mice were obtained from Harlan Laboratories/Envigo and were maintained at the Glasgow University Central Research Facility. Mice were kept for one week prior to commencement of procedures to allow for acclimatisation and were maintained in specific pathogen-free conditions. Mice were treated between 8–9 weeks of age and were age- and sex- matched for each study. All experiments received local ethical approval and were performed in accordance with the animal care and welfare protocols approved by the Animal Welfare and Ethical Review Board at the University of Glasgow and were carried out under the auspices of a UK Home Office Project Licence.

### Aldara Timecourse Model

8–9 week old C57BL/6 female mice were treated with a quarter sachet (≈62.5 mg) of Aldara™ (Meda AB) cream containing 5% Imiquimod or an equivalent volume of control cream (Boots Aqueous Cream/10% Vaseline Lanette Cream) at each treatment point. Cream was applied to the shaved dorsal skin near the base of the tail and for time-points exceeding 24 hours mice were weighed daily. Mice were euthanised at 4 hours, 12 hours and 24 hours after the first treatment, or 24 hours after the 3rd and 5th treatment (4 h, 12 h, 1d, 3d, 5d groups respectively) with an increasing concentration of CO2.

### Tissue Collection for RNA and protein

Blood was collected from mice immediately after euthanasia using a syringe flushed with 0.5 M EDTA. In brief, PBLs were obtained by separating the plasma and cellular compartments using centrifugation at 300 g for 10 minutes. Plasma was removed from the sample and red cell lysis treatment was performed for 10 minutes with ammonium chloride (STEMCELL Technologies). PBLs were re-suspended in buffer RLT (Qiagen) and stored at −80 °C.

Following blood collection, mice were perfused with 20 ml PBS through the left ventricle to clear the tissues. Following perfusion tissues (skin, lungs, liver and brain) were collected, placed in cryovials and snap frozen in liquid nitrogen. Tissues were stored at −80 °C until RNA extraction was performed.

### Tissue Collection for FFPE Sections

The left hemisphere of the brain was collected from mice after euthanasia and placed into 10% neutral buffered formalin for 24–48 hours before being transferred to 70% ethanol prior to tissue processing. Samples were processed using a KOS microwave HistoSTATION (Milestone SRL) before being embedded in paraffin wax.

### Tissue Collection for Mass Spectrometry

After opening the right atrium, whole blood was collected in syringes flushed with lithium-heparin PBS before being placed into 1.5 ml Eppendorf containing 40 ul lithium-heparin PBS (8U lithium-heparin). Mice were subsequently perfused with 20 ml lithium-heparin PBS and brains were collected, placed in cryovials and snap frozen in liquid nitrogen. Plasma was immediately collected from whole blood using two rounds of centrifugation at 10,000 g for 5 minutes. Both plasma and brains were stored at −80 °C until shipping on dry ice.

### QRT-PCR

PBL and tissues were collected as described. Under RNase-free conditions, tissues were homogenized using the TissueLyser LT (Qiagen) in 1 ml of Qiazol® (Qiagen). RNA was isolated from PBL and tissue supernatants using the RNeasy Mini Kit (Qiagen) with DNase I (Qiagen) on-column treatment as per the manufacturer’s instructions. RNA quality was assessed using an Agilent Bioanalyzer (Agilent Technologies) at Glasgow Polyomics. RNA was converted to cDNA using High-Capacity RNA-to-cDNA Kit (Thermo Fisher Scientific) as per the manufacturer’s instructions. cDNA concentration was standardized by using an equal quantity of RNA (measured using a Nanodrop). qRT-PCR was performed using QuantStudio7 (ThermoFisher Scientific) qRT-PCR machines. PerfeCTa SYBR Green FastMix with ROX reference dye (QuantaBio) was used for all reactions. Samples were run in triplicate on 384 Well PCR Plates (Starlab) and were quantified using a 6-point standard curve. Analysis was performed following a series of quality control steps and gene expression was calculated relative to the housekeeping gene, Tbp.

### LegendPlex Protein Assay

Protein was extracted from perfused brain tissue using N-PER neuronal extraction reagent (Thermo Fisher Scientific) as per manufacturer’s instructions. Homogenate was centrifuged at 10,000 g for 10 minutes at 4 C and supernatant was collected, snap frozen on dry ice and stored at −80 °C prior to use. Protein quality was assessed using western blot against the housekeeping protein GAPDH to ensure degradation had not occurred. Protein concentration was determined using Pierce BCA Protein Assay Kit (ThermoFisher Scientific, MA, USA) as per manufacturer’s instructions. Cytokine and chemokine expression in plasma and brain supernatant was measured using the LegendPlex protein assay (BioLegend). In brief, beads were incubated with protein extract for 2 hours at 600 rpm on a plate shaker. Beads were then conjugated with streptavidin-phycoerythrin (SA-PE) for 30 minutes and two washes were performed prior to sample reading using a BD LSR-II flow cytometer (BD Biosciences). Samples were differentiated on the basis of bead size and APC fluorescence, while protein quantity was determined using SA-PE fluorescence calibrated to a standard curve.

### Mass Spectrometry

Plasma and brain samples were sent to the Biomarker and Drug Analysis Core Facility at the University of Dundee (http://medicine.dundee.ac.uk/biomarker-and-drug-analysis-core-facility) for mass spectrometry analysis. Briefly, samples were homogenized and subjected to solid phase extraction using in-house strong cation exchange SpinTip. Eluents were dried and then reconstituted in 25 μL elution solution. Liquid chromatography coupled with tandem mass spectrometry was perfomed using a HILIC colum (Intersil, GL Sciences) and a Thermo Quantum Ultra mass spectrometer (ThermoFisher Scientific). The lower limit of quantification was 1.57 ng/mL. Full mass spectrometry methods are provided in the supplementary information.

### Immunohistochemistry

Brains were cut to 5 μm sagittal sections using a Shandon Finesse 325 Microtome (Thermo Fisher Scientific) and were stained for Iba1, GFAP or appropriate isotypes and negative controls. Three non-consecutive slides were stained for each cell type from each mouse brain. Blinded slides were sent to the Beatson Institute (University of Glasgow) to be scanned. Iba1 and GFAP hippocampal area quantification was carried out using ImageJ software.

### Statistical analysis

All statistical analysis was performed in GraphPad Prism 7 software. Gene and protein expression data were log transformed prior to parametric tests. Student’s t-test or one-way ANOVA (with post-hoc tests) were used to test for significant differences between groups as indicated in the figure legend. If multiple genes or time points were tested in the same experiment, Bonferroni’s multiple correction testing was applied to account for the increased probability of type I errors. Linear regression analysis was performed to determine the association between plasma and brain imiquimod levels. A p-value of <0.05 was considered significant.

### Data availability

All data generated or analysed during this study are included in this published article (and its Supplementary Information files).

## Electronic supplementary material

Supplementary Information
