# Supplementary material for: The Systemic Response to Topical Aldara Treatment is Mediated Through Direct TLR7 Stimulation as Imiquimod Enters the Circulation
Source: Sci Rep. 2017 Nov 29;7:16570. doi: 10.1038/s41598-017-16707-5 (PMC5707416; doi:10.1038/s41598-017-16707-5)
Supplement: Supplementary file 1 — Supplementary Information [file 41598_2017_16707_MOESM1_ESM.pdf]

The Systemic Response to Topical Aldara Treatment is Mediated Through Direct TLR7 Stimulation as Imiquimod Enters the Circulation

Louis Nerurkar[1], Alison McColl[1], Gerard Graham[1], and Jonathan Cavanagh[2]*

[1]University of Glasgow, Institute of Infection, Immunity & Inflammation, Glasgow, G12 8TA, Scotland
[2]University of Glasgow, Institute of Health & Wellbeing, Glasgow, G12 8TA, Scotland
*jonathan.cavanagh@glasgow.ac.uk

Supplementary Information

Mass Spectrometry methods

Quantification of imiquimod in brain and plasma from mice treated with topical Aldara 5% cream was based on a stable isotope dilution LC-MS/MS method described as follows. For brain, the right hemisphere of brains were weighed and 5mL acetonitrile and 2mL 1% acetic acid (in water) were added to each sample. Homogenisation was carried out using MSE SoniPrep 150 Ultrasonicator (15 seconds at full power). 50uL internal standard (50ng/mL d7-imiquimod in DMSO) was then added in each homogenate. The samples were then subjected to further homogenisation (5 seconds x 3 times) to ensure that an equilibrium is reached. One tenth (725uL) of homogenate was centrifuged at 13k x g for 10min and the supernatant was subjected to solid phase extraction. For plasma, 25uL plasma samples, 25 uL internal standard (10ng/mL d7-imiquimod in DMSO), 0.5mL acetonitrile and 0.2mL 1% acetic acid in water were combined and left on ultrasound bath for 5 min at room temperature. The sample was centrifuged at 13k x g for 10min and the supernatant was subjected to solid phase extraction.

Solid phase extraction was carried out using in-house strong cation exchange SpinTip, which were preconditioned with 2mL 1% acetic acid in methanol, equilibrated with 2mL 1% acetic acid in 60% acetonitrile. After sample loading, the SpinTip was washed with 1mL 100% acetonitrile. Elution was carried out by 50uL 8.7% ammonia in methanol. The eluents were dried under vacuum and reconstituted in 25uL elution solution (ACN: MeOH: Water (8:1:1)). All processes were performed in a desktop centrifuge at 3000 x g.

LC-MS/MS analysis: Imiquimod was resolved on a HILIC column (Intersil, GL Sciences, 2x 150mm) using a 5 min gradient (0min at 0% B, 2.5min to 50%B, 2.7min to 100%B, 4min to 100%B, 4.2min to 0%B and 5min to 0%B), where buffer A is 0.1% formic acid in acetonitrile and buffer B is 0.1% formic acid in water. The flow rate is 0.65mL/min. IonMax was interfaced between the LC and a Thermo Quantum Ultra mass spectrometer (Thermo). The spray voltage is set at 3.5kv, capillary temperature at 270◦C, Sheath gas pressure at 15, aux gas pressure at 5 and skimmer offset at -1. Two transitions per compound were monitored. For Imiquimod, the transitions were 241-185 (for quantification), and 241-168 (for qualification); for d7-imiquimod, the transitions were 248-185, and 248-168. The resolution at Q1 and Q3 were set at 0.7.The collision gas of argon is set at 1.5mTorr. The lower limit of quantification is 1.57ng/mL (<15% of imprecision and in accuracy) and the spike-in recovery was 87%.

Supplementary Figures

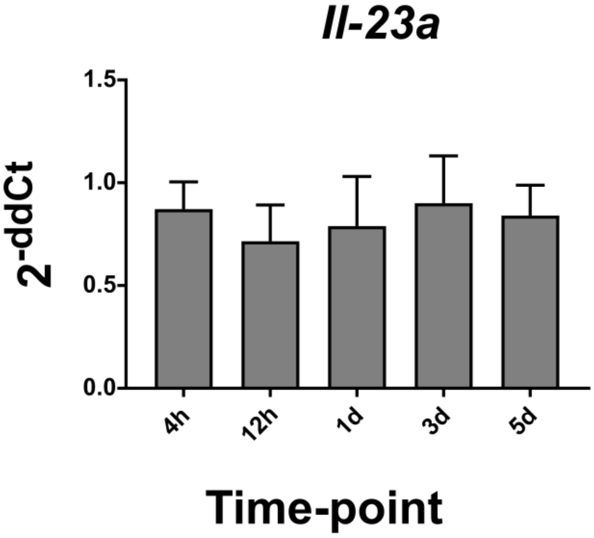

Supplementary Figure 1. Topical Aldara application does not induce an *Il-23a* response within the brain

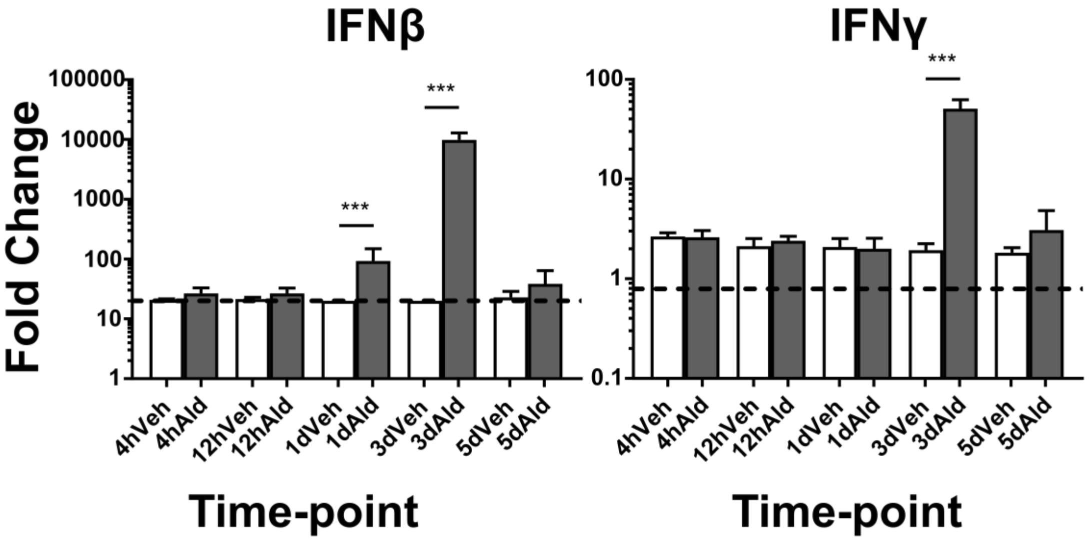

Supplementary Figure 2. Topical Aldara application induces an IFN protein response in the brain.

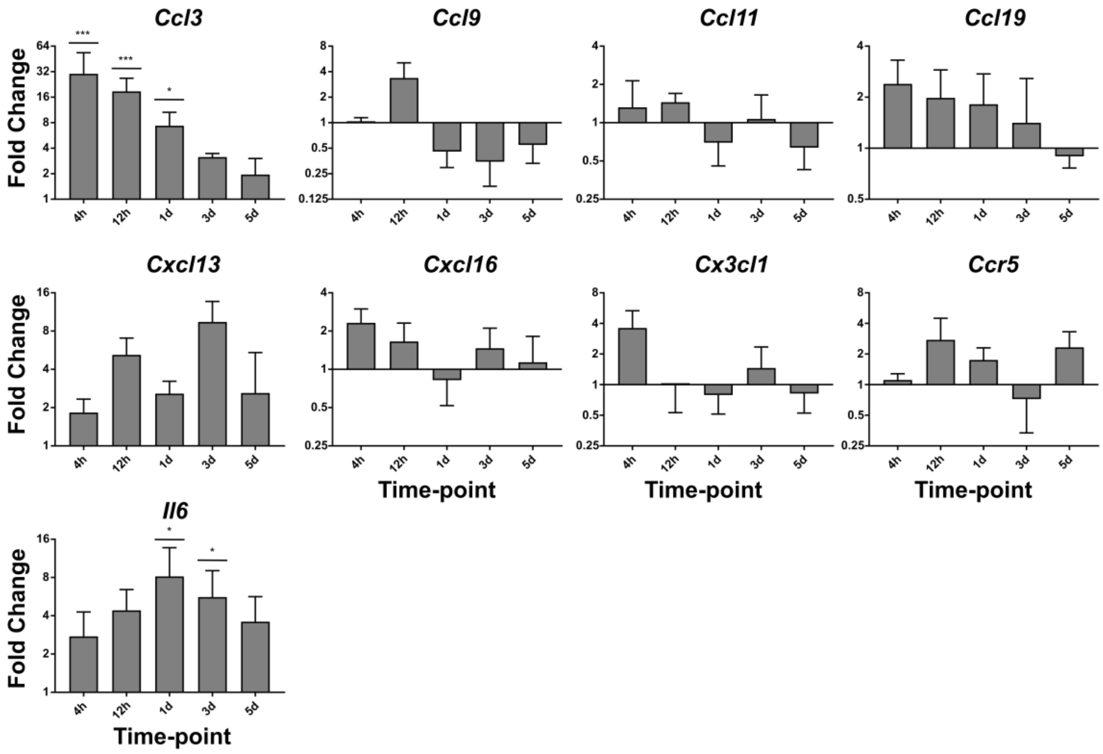

Supplementary Figure 3. Topical Aldara induces a limited chemokine and cytokine transcriptional response in the skin of treated mice.

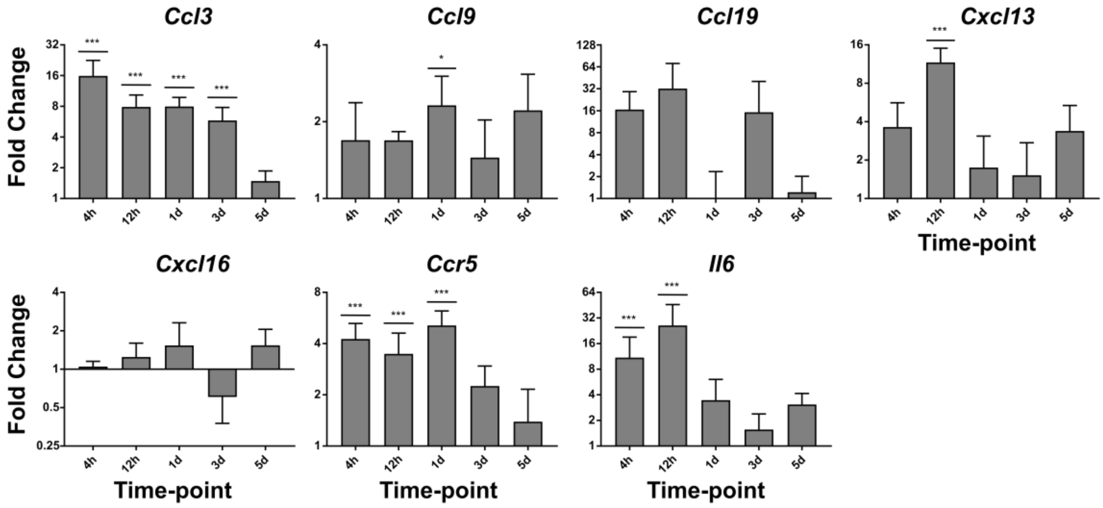

Supplementary Figure 4. Topical Aldara induces a chemokine and cytokine transcriptional response in PBLs of treated mice.

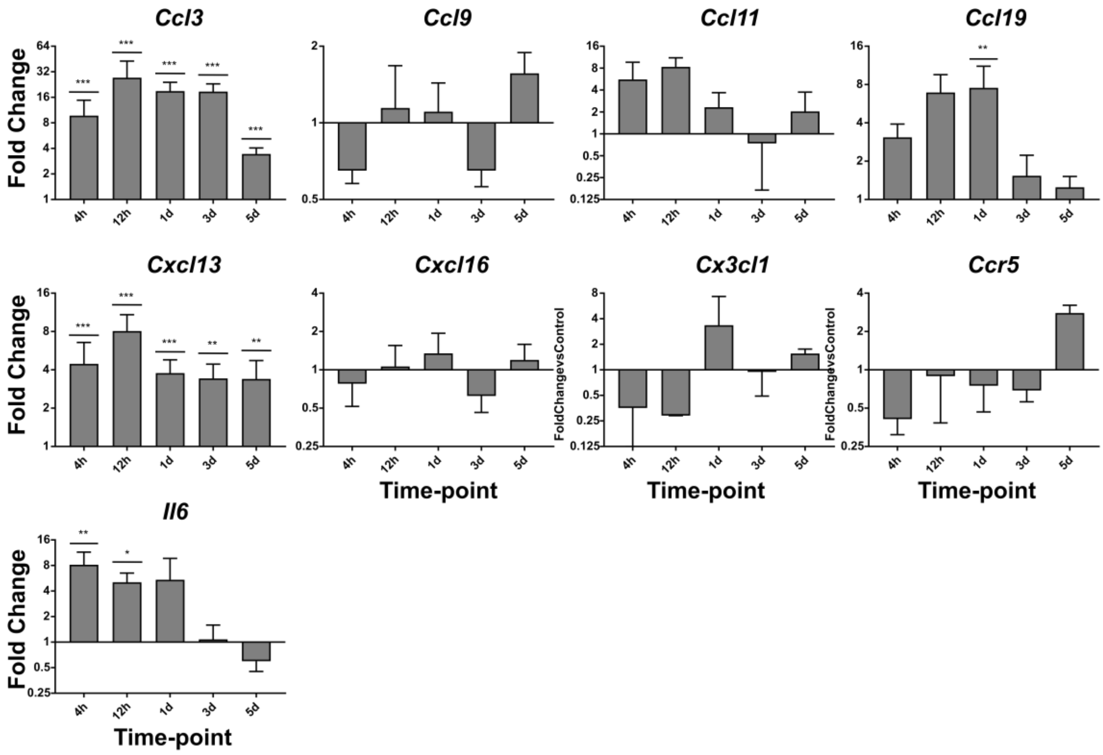

Supplementary Figure 5. Topical Aldara induces a chemokine and cytokine transcriptional response in the liver of treated mice.

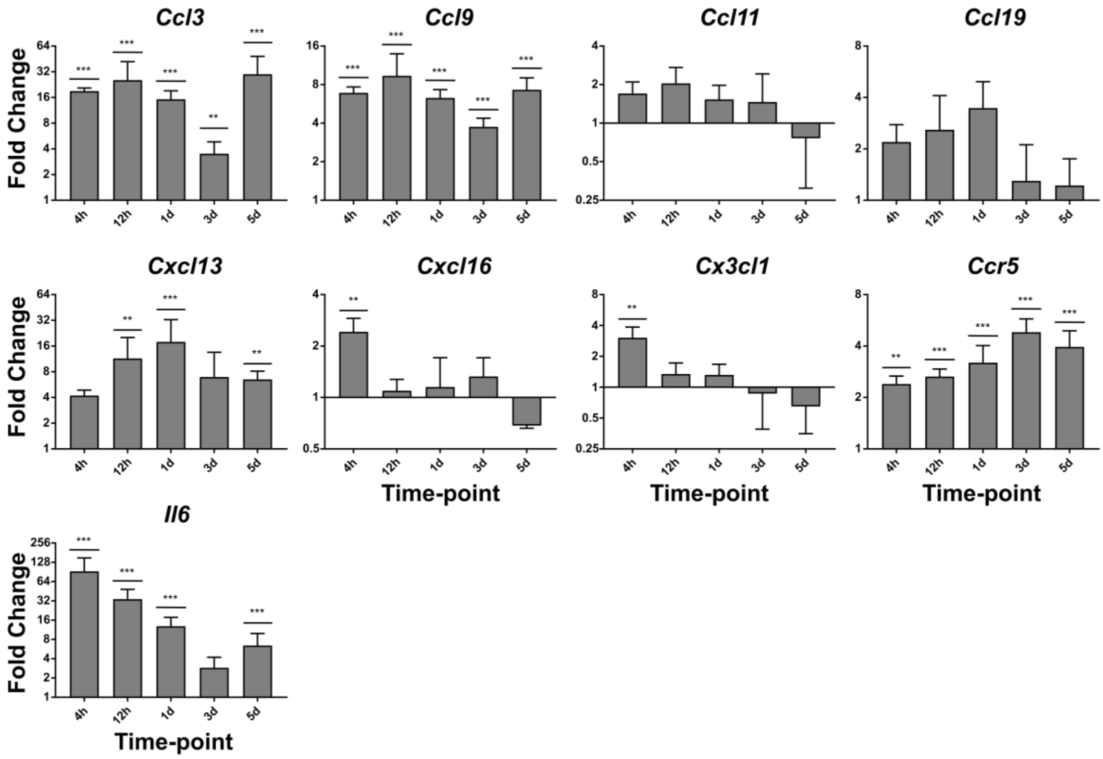

Supplementary Figure 6. Topical Aldara induces a prolonged chemokine and cytokine response in the lungs of treated mice.

Supplementary Figure Legends

Supplementary Figure 1. Topical Aldara application does not induce an *Il-23a* response within the brain

QRT-PCR analysis was performed on RNA from perfused brain samples from mice treated daily with Aldara or control cream. Samples were collected 4hr, 12hr, 1d, 3d and 5d after the initial treatment. Expression values were normalised to housekeeping gene *Tbp* and analysed using the delta delta Ct method. Data are presented as mean (+/- SD) 2^{-ddCt} vs respective controls. N = 4 mice per group. Data were log transformed and significance was determined using one-way ANOVA with post-hoc Student's t-test with Bonferroni's multiple testing correction. A p value of less than 0.05 was considered significant.

Supplementary Figure 2. Topical Aldara application induces an IFN protein response in the brain.

Perfused brain samples from mice treated daily with Aldara or control cream were collected 4hr, 12hr, 1d, 3d and 5d after the initial treatment. Biolegend LegendPlex protein array was performed. Data are presented as absolute protein values. N=4 mice per group. Data were log transformed and significance was determined using one-way ANOVA with post-hoc Student's t-test with Bonferroni's multiple testing correction. * = P<0.05; ** = P<0.01; *** = P<0.001.

Supplementary Figure 3. Topical Aldara induces a limited chemokine and cytokine transcriptional response in the skin of treated mice.

QRT-PCR analysis was performed using perfused skin samples from mice treated daily with Aldara or control cream. Samples were collected 4hr, 12hr, 1d, 3d and 5d after the initial treatment. The expression profile of 7 chemokines, 1 chemokine receptor and 1 cytokine was assessed. Expression values were normalised to housekeeping gene *Tbp*. Data are presented as mean (+/- SD) fold changes vs respective controls. N = 4 mice per group. Data were log transformed and significance was determined using one-way ANOVA with post-hoc Student's t-test with Bonferroni's multiple testing correction. * = P<0.05; *** = P<0.001.

Supplementary Figure 4. Topical Aldara induces a chemokine and cytokine transcriptional response in PBLs of treated mice.

QRT-PCR analysis was performed using PBLs obtained from the whole blood of mice treated daily with Aldara or control cream. Samples were collected 4hr, 12hr, 1d, 3d and 5d after the initial treatment. The expression profile of 5 chemokines, 1 chemokine receptor and 1 cytokine was assessed. Ccl11 and Cx3cl1 were also measured but expression was too low to quantify (data not shown). Expression values were normalised to housekeeping gene *Tbp*. Data are presented as mean (+/- SD) fold changes vs respective controls. N = 4 mice per group. Data were log transformed and significance was determined using one-way ANOVA with post-hoc Student's t-test with Bonferroni's multiple testing correction. * = P<0.05; *** = P<0.001.

Supplementary Figure 5. Topical Aldara induces a chemokine and cytokine transcriptional response in the liver of treated mice.

QRT-PCR analysis was performed using perfused liver samples from mice treated daily with Aldara or control cream. Samples were collected 4hr, 12hr, 1d, 3d and 5d after the initial treatment. The expression profile of 7 chemokines, 1 chemokine receptor and 1

cytokine was assessed. Expression values were normalised to housekeeping gene *Tbp*. Data are presented as mean (+/- SD) fold changes vs respective controls. N = 4 mice per group. Data were log transformed and significance was determined using one-way ANOVA with post-hoc Student's t-test with Bonferroni's multiple testing correction. * = P<0.05; ** = P<0.01; *** = P<0.001.

Supplementary Figure 6. Topical Aldara induces a prolonged chemokine and cytokine response in the lungs of treated mice.
QRT-PCR analysis was performed using perfused lung samples from mice treated daily with Aldara or control cream. Samples were collected 4hr, 12hr, 1d, 3d and 5d after the initial treatment. The expression profile of 7 chemokines, 1 chemokine receptor and 1 cytokine was assessed. Expression values were normalised to housekeeping gene *Tbp*. Data are presented as mean (+/- SD) fold changes vs respective controls. N = 4 mice per group. Data were log transformed and significance was determined using one-way ANOVA with post-hoc Student's t-test with Bonferroni's multiple testing correction. ** = P<0.01; *** = P<0.001.
